# Prognostic Value of Posteromedial Cortex Deactivation in Mild Cognitive Impairment

**DOI:** 10.1371/journal.pone.0001104

**Published:** 2007-10-31

**Authors:** Jeffrey R. Petrella, Steven E. Prince, Lihong Wang, Caroline Hellegers, P. Murali Doraiswamy

**Affiliations:** 1 Alzheimer Imaging Research Laboratory and Brain Imaging and Analysis Center, Department of Radiology, Duke University Medical Center, Duke University, Durham, North Carolina, United States of America; 2 Department of Psychiatry, Duke University Medical Center, Duke University, Durham, North Carolina, United States of America; 3 Department of Medicine (Geriatrics), Duke University Medical Center, Duke University, Durham, North Carolina, United States of America; 4 Center for Study of Aging, Duke University Medical Center, Duke University, Durham, North Carolina, United States of America; Claremont Graduate University, United States of America

## Abstract

**Background:**

Normal subjects deactivate specific brain regions, notably the posteromedial cortex (PMC), during many tasks. Recent cross-sectional functional magnetic resonance imaging (fMRI) data suggests that deactivation during memory tasks is impaired in Alzheimer's disease (AD). The goal of this study was to prospectively determine the prognostic significance of PMC deactivation in mild cognitive impairment (MCI).

**Methodology/Principal Findings:**

75 subjects (34 MCI, 13 AD subjects and 28 controls) underwent baseline fMRI scanning during encoding of novel and familiar face-name pairs. MCI subjects were followed longitudinally to determine conversion to AD. Regression and analysis of covariance models were used to assess the effect of PMC activation/deactivation on conversion to dementia as well as in the longitudinal change in dementia measures. At longitudinal follow up of up to 3.5 years (mean 2.5±0.79 years), 11 MCI subjects converted to AD. The proportion of deactivators was significantly different across all groups: controls (79%), MCI-Nonconverters (73%), MCI-converters (45%), and AD (23%) (p<0.05). Mean PMC activation magnitude parameter estimates, at baseline, were negative in the control (−0.57±0.12) and MCI-Nonconverter (−0.33±0.14) groups, and positive in the MCI-Converter (0.37±0.40) and AD (0.92±0.30) groups. The effect of diagnosis on PMC deactivation remained significant after adjusting for age, education and baseline Mini-Mental State Exam (p<0.05). Baseline PMC activation magnitude was correlated with change in dementia ratings from baseline.

**Conclusion:**

Loss of physiological functional deactivation in the PMC may have prognostic value in preclinical AD, and could aid in profiling subgroups of MCI subjects at greatest risk for progressive cognitive decline.

## Introduction

Accumulating evidence suggests that synaptic dysfunction precedes neuronal death in the earliest stages of Alzheimer's disease (AD) [1], [2], [3]. Hence studies of early functional brain changes in patients at risk for AD offer promise to compliment information from other biomarkers such as cognitive tests, spinal fluid changes, metabolic changes, and medial temporal volume loss [4], [5], [6]. Functional magnetic resonance imaging (fMRI) studies during episodic memory encoding tasks in AD have demonstrated decreased medial temporal activation [7], [8], [9], [10], [11], [12]. However, medial temporal lobe fMRI results in mild cognitive impairment (MCI) have been equivocal, with some studies demonstrating increased activation [13], [14] and others decreased activation [9], [14], [15], [16] possibly due to sampling differences.

The posteromedial cortex (PMC), an archectonically discrete zone comprised of the posterior cingulate, precuneus and retrosplenial cortex, is hypothesized to be part of a network called the “default network”. In normal younger subjects there is functional deactivation (decreased activation) in this network during a broad range of cognitively challenging tasks [17]. Prior FDG PET studies have shown that the PMC region is one of the earliest to be affected in subjects at risk for AD [18]. Recent cross-sectional fMRI studies have extended these findings by showing that task-related functional PMC deactivation is reduced in AD and MCI [14], [19], [20]. At baseline, there is a continuum, with normal controls demonstrating the most efficient deactivation, AD subjects the least efficient deactivation, and MCI subjects falling in between [21].

Although regions outside the PMC, including the frontal and medial temporal lobes, have been implicated in previous activation studies of MCI subjects, findings in the PMC are the strongest in studies looking at both activation and deactivation [14], [21]. Because our previous crossectional data in AD, amnestic MCI and control subjects revealed the most robust fMRI changes in the PMC [21], we focused on this region as the best possible predictor of cognitive decline in amnestic MCI subjects in the present study. To our knowledge no prior study has tested the prognostic value of PMC deactivation in MCI.

## Methods

### Subjects

The study was approved by our Institutional Review Board and conducted in compliance with the Health Insurance Portability and Accountability Act. Subjects satisfying entry criteria were recruited from the local community via advertisements and referrals. All subjects gave written informed consent prior to participation. When appropriate, consent was also obtained from an informant and/or legal guardian consistent with current practices for AD research [22]. A total of 98 subjects (mean age±SD = 73.4±7.2, 49M, 49F, 20 mild AD, 44 MCI, 34 controls) were recruited and underwent fMRI scanning. Of these subjects, 23 (7 mild AD, 10 MCI, 6 controls) were eliminated because of technical factors or insufficient quality of fMRI scan data (see Exclusion Criteria), leaving 75 subjects for analysis (mean age±SD = 72.9±7.2, 37M, 38F, 13 mild AD, 34 MCI, 28 controls) (Table 1).

**Table 1:** Demographics and summary statistics.

	Control	MCI	AD
Age*	71.96 (4.94)	73.64 (8.48)	71.37 (6.80)
M/F ratio*	14/14	15/18	7/6
Education†	16.32 (2.79)	15.03 (2.52)	12.69 (2.25)
MMSE‡	28.25 (1.40)	26.82 (1.74)	24.62 (2.43)
WMSIII Logical Memory -delayed recall‡	12.71(2.48)	9.48 (3.30)	5.07 (2.02)
WMSIII Visual Reproduction-delayed recall‡	13.36 (2.86)	9.58 (3.11)	5.31 (1.44)
Beck II Depression Inventory*	5.32 (4.57)	5.12 (3.92)	5.15 (3.87)
Hachinski Vascular Dementia Scale*	1.93 (1.11)	2.29 (1.30)	1.75 (2.00)
% Novel correct score on fMRI task‡ (of 30 trials)	0.71 (0.15)	0.58 (0.18)	0.46 (0.09)
% Familiar correct score on fMRI task† (of 30 trials)	0.96 (0.08)	0.89 (0.17)	0.80 (0.18)
% Novel and familiar correct score on fMRI task‡	0.84 (0.10)	0.74 (0.15)	0.63 (0.12)
% Non-recorded responses (of 60 trials)†	0.02 (0.02)	0.06 (0.09)	0.12 (0.16)

* **= NS,**

† **= p<0.05,**

‡ **= p<0.001**

*Gender tested via Chi square, other categories tested via ANOVA*

Unless otherwise noted, means (SD) are listed.

### Entry Criteria

All subjects were fluent in English. Subjects underwent diagnostic evaluations including a clinical interview and focused neurological and mental status exam, review of medical history, demographic inventory (age, gender, education) and brain MRI scan. Subjects then underwent neuropsychological testing including the California Verbal Learning Test (CVLT) II [23], Logical Memory and Visual Reproduction tests from the Wechsler Memory Scale (WMS) III [24], the Mini-Mental State Exam (MMSE) [25], a Clinical Dementia Rating (CDR)[26] interview with patient and informant, Beck-II Depression Scale [27], and Rosen-modified Hachinski vascular dementia rating scale [28].

### Inclusion Criteria

Mild AD subjects met the following criteria: 1) a history of progressive cognitive worsening for at least 1 year; 2) a Rosen-modified Hachinski score of ≤4; 3) National Institute of Neurological Disorders and Stroke (NINCDS-ADRDA) [29] criteria for probable AD; 4) an MMSE score of ≥20, 5) a CDR global score of 1.0 (mild dementia), with a memory score of at least 1.0.

MCI (amnestic type) subjects met the following criteria: 1) recent history of symptomatic worsening in memory; 2) a Rosen-modified Hachinski score of ≤4; 3) impaired delayed recall memory performance; 4) MMSE score of 22–30; 5) CDR global score of 0.5, with 0.5 or greater on the memory score; 6) did not meet NINDS or DSM-IV criteria for dementia, 7) normal or near normal independent function; and 8) absence of other factors that might have better explained memory loss, for example, current major depression. These criteria were derived from prior research [30]. Five subjects classified as MCI did not have an informant and hence their baseline CDR score was inferred using other information for classification purposes.

Controls met the following criteria: 1) a Rosen modified Hachinski score of ≤4; 2) near normal memory performance on delayed recall; 3) MMSE score of 25–30; 4) a CDR global score and memory score of 0; and 5) normal independent function. Controls did not meet NINCDS-ADRDA criteria for AD or DSM-IV criteria for dementia and were judged to have normal cognition.

### Exclusion Criteria

Subjects were excluded on the basis of the following criteria: 1) uncontrolled depression or other significant psychiatric or neurological illness such as recent stroke, 2) taking psychoactive medications known to substantially affect memory, 3) standard contraindications to MRI 4) technical difficulties that prevented the completion of successful anatomical imaging or at least 2 of 3 functional MRI runs, or both, 5) excessive motion during the functional MRI exam in excess of 5mm in any of three orthogonal directions, determined by center of mass plots, and 6) inability to adequately monitor subject behavioral responses while in the scanner, evidenced by greater than 50% non-responses.

### Longitudinal Follow-up

All MCI subjects who completed fMRI scans successfully were invited to return for clinical evaluation every 6 months after baseline until they converted to AD, or until the end of the study period. The follow-up evaluations at each visit included informant interviews, neuropsychological testing and CDR ratings. During follow up, MCI subjects were reclassified as MCI-Converter or MCI-Nonconverter based on whether they were subsequently diagnosed with dementia. The clinical diagnosis of dementia was triggered by change in Clinical Dementia Rating (CDR) scale score from 0.5 to 1.0, and confirmed by physician evaluations and neuropsychological tests. Change in CDR-SOB score was available in 27 of 34 MCI subjects (1 lost to follow-up, 5 did not have a baseline CDR, and 1 did not have a follow-up CDR score, in the latter two situations because of unavailability of an informant). In subjects in whom CDR was not available, a diagnosis at start and endpoint was made using all other clinical information. The study period of five years allowed for only up to 3.5 years of follow-up due to initial time for study start up and enrollment.

### Imaging and Image Analysis

All subjects underwent baseline fMRI scanning at 4.0T (GE Medical Systems, Milwaukee, WI) during encoding of novel and familiar face-name pairs, presented in a blocked experimental design using an MR compatible goggle system (Resonance Technology, Northridge, CA). Sixty novel and two familiar face-name pairs were presented in 3 runs, for 6 minutes, 50 seconds per run. Behavioral responses were monitored with a fiber-optic button box within the scanner, with non-responses counted as incorrect responses. The paradigm, MR scanning parameters, and individual subject image analysis are described in greater detail in previous reports [15], [21]. Briefly, anatomic and functional scans were acquired over the same 44 continuous slice locations in the coronal plane with the functional images measuring 3.75-mm-thick and consisting of a time series of 164 T2*-weighted isotropic image volumes (inverse spiral echo-planar imaging [EPI]; repetition time [TR] 2500 ms; echo time [TE] 31 ms; flip angle [FA] 60°; matrix 64×64; field of view [FOV] 240 mm). Images were processed using Statistical Parametric Mapping (SPM2) software [31]. The functional time series underwent slice timing correction, spatial smoothing, global intensity scaling, and normalization to a standardized brain template. The magnitude of blood-oxygen-level-dependent (BOLD) signal changes were assessed on a voxelwise basis using a general linear model approach. A contrast map was created for each subject, depicting mean signal magnitude change between the novel and familiar encoding conditions across the entire brain.

**Figure 1:**
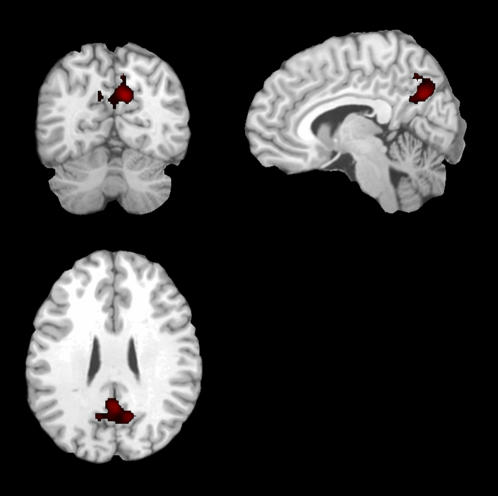
Posteromedical cortex (PMC), denoted in red and overlaid on a canonical T1-weighted brain template image in three orthogonal views, was used as an apriori functional region of interest. In this region parameter estimates for activation magnitude showed a lesser-to-greater activation from Control, to MCI, to AD subjects.

An apriori, hypothesis-driven functional region of interest (fROI) was chosen on the basis of prior data [21] demonstrating significant cross-sectional differences in activation from healthy elderly controls to AD subjects. This fROI was located in the posteromedial cortical (PMC) region (Fig 1), which is defined as the precuneus, posterior cingulate, and retrosplenial cortices. This fROI was then applied to the contrast maps of each individual subject, and the mean activation magnitude parameter estimate in the PMC was determined.

### Statistics

Statistical analysis was performed with SPSS software (version 12.2, 2004, SPSS Inc., Chicago, IL). For each group, summary statistics were generated for demographic and behavioral variables, as well as performance on fMRI task (percentage correct novel and familiar trials), and number of non-recorded responses. An analysis of variance (ANOVA) was performed for all continuous variables, and a chi-squared analysis was performed for gender.

For statistical analysis of the imaging data, subjects were classified into the following four groups, as specified above: mild AD, MCI-Converter, MCI-Nonconverter, and controls. Activation in the PMC was considered both a nominal and continuous variable. For treatment as a nominal variable, the category “deactivator” was used to denote an activation magnitude less that zero, and “activator” was used to denote activation magnitude greater than or equal to zero [19], [32]. A logistic regression model was applied using activator/deactivator status as the dependent variable, and group as an ordinal independent variable, with age, education and MMSE score as covariates. For treatment as a continuous variable, an analysis of covariance (ANCOVA) model was applied using mean activation magnitude parameter estimate in the PMC as the dependent variable, and group as an independent variable, with age, education and MMSE score as covariates, and Fisher's post hoc testing. Change in cognitive status was also examined as a continuous variable in the MCI subjects, using change in CDR-SOB (sum of boxes) score and change in MMSE score. These were calculated by subtracting the baseline score from the most recently available score. Separate Pearson correlations were performed using mean activation in the PMC as the dependent variable, and change in CDR-SOB score, and change in MMSE score as the independent variables. A p-value of <0.05 was considered statistically significant. ANCOVA and logistic regression analyses were also performed after adjusting for behavioral performance on the fMRI task using novel correct and familiar correct measures.

## Results

### Demographics and Behavioral Variables

Demographics and behavioral test scores with summary statistics for the 75 subjects are given in Table 1. Age, gender, depression inventory and ischemia rating scale scores were not significantly different among groups, however as expected, years of education, MMSE and memory scores were significantly different among groups.

### Longitudinal Clinical Follow-up

The mean clinical follow-up for MCI subjects was 2.50 (0.79) years. Eleven (33%) converted during the study period with mean time-to-conversion of 2.46 (0.70) years. For the nonconverters, mean follow-up time was 2.52 (0.84) years. There was no statistically significant difference between mean time to conversion in MCI-Converters and mean follow-up time in MCI-Nonconverters (p = 0.84) over the course of this 5-year study.

### PMC Deactivation

**Figure 2:**
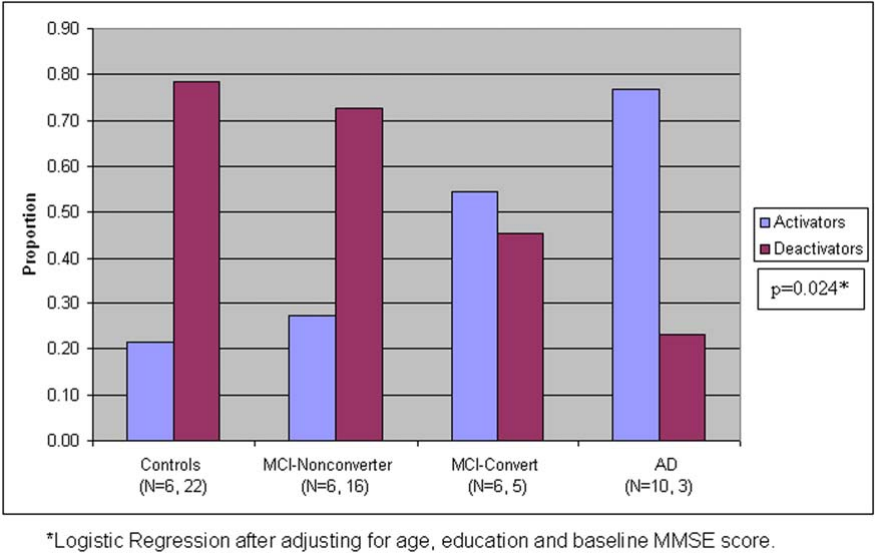
Bar graph demonstrates the proportion of activators and deactivators among the four diagnostic groups. The proportion of deactivators was significantly different (p<0.05) across diagnostic groups after adjusting for age, education and baseline MMSE score, with significantly fewer deactivators with increasing cognitive impairment grouping.

Logistic regression demonstrated that the proportion of deactivators was significantly different (p<0.05) across diagnostic groups after adjusting for age, education and baseline MMSE score: controls (79%), MCI-Nonconverters (73%), MCI-converters (45%), and AD (23%), with significantly fewer deactivators with increasing cognitive impairment grouping (Figure 2). The main effects of age, education and baseline MMSE score were not significant in the model.

**Figure 3:**
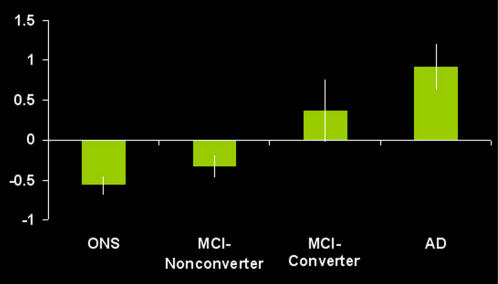
Bar graph demonstrates activation magnitude parameter estimate in the PMC region (taken from Figure 1), demonstrating a continuum from control, to MCI-Nonconverter, to MCI-Converter, to AD. There were statistically significant (p<0.05) differences between all groups with the exception of the control and MCI-Nonconverter group, and the AD and MCI-Converter group. Note the overall pattern of negative activation magnitude in the Control and MCI-Nonconverter groups, and positive activation magnitude in the AD and MCI-Converter groups.

**Figure 4:**
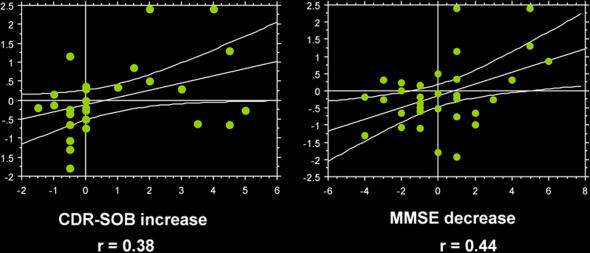
Scatterplots demonstrate activation magnitude parameter estimate in the PMC region (on the y-axis) significantly (p<0.05) correlated with longitudinal change in cognitive measures (increase in CDR-SOB, decrease in MMSE) in the MCI group.

Mean (SD) PMC regression coefficient activation magnitude for all four groups revealed a continuum from Controls, to MCI-Nonconverters, to MCI-Converters, to AD with negative magnitude in the Control and MCI-Nonconverter groups, and positive activation magnitude in the MCI-Converter and AD groups: Controls -0.57 (0.12); MCI-Nonconverters -0.33 (0.14), MCI-converters 0.37 (0.40), and AD 0.92 (0.30) (Figure 3). The ANCOVA revealed a significant group main effect, independent of age, education and MMSE score (p<0.05). The main effects of the latter three variables were not significant in the ANCOVA model. ANOVA post hoc testing revealed significant differences between all groups, except the AD and MCI-Converters, and Controls and MCI-Nonconverters. The correlation between PMC activation magnitude and change in CDR-SOB score was 0.38 (p<0.05), and change in MMSE score 0.44 (p<0.05) (Figure 4). ANCOVA and logistic regression analyses after adjusting for behavioral performance remained significant (p<0.05).

## Discussion

While most fMRI studies in dementia have focused on positive activations, or increases in task-related signal, recent studies in AD have examined task-related deactivations, defined as a decrease in signal during an active vs. passive task. Previous cross-sectional fMRI studies in AD and MCI subjects examined deactivation in the PMC region during memory encoding tasks [19], [20], [21]. Using a word encoding task in 23 AD subjects, 32 young, and 27 older controls, Lustig et al. first demonstrated loss of deactivation in the medial parietal/posterior cingulate region in AD and elderly control subjects, compared to young subjects, with positive activations in the AD group [19]. Moreover, using an exploratory approach, Lustig et al. showed the PMC region to be the site of the largest groups differences between AD and elderly controls found throughout the brain. Rombouts et al. furthered these findings in a study of 28 MCI subjects, 18 subjects with AD and 41 elderly controls [20]. They demonstrated deactivation in the PMC in the elderly control group during a face encoding task. MCI subjects showed smaller regions of deactivation in the PMC and medial frontal cortex, compared to controls and larger regions compared to AD subjects. Petrella et al. confirmed and furthered these findings, by demonstrating a correlation between cognitive performance and PMC activation magnitude across the spectrum of AD, MCI and elderly control subjects [21]. These findings prompted us to examine the prognostic significance of deactivation in this region.

The following findings emerged from our study: 1) MCI subjects who convert to AD have impaired PMC deactivation compared to those who do not convert, independent of age, education and baseline MMSE score. Baseline PMC deactivation also correlates with change from baseline in CDR-SOB; 2) Group membership, and therefore disease status, is a more robust predictor of PMC deactivation than age, education or MMSE score; 3) PMC activation differences appear opposite in sign, with MCI-nonconverter group demonstrating negative mean activation (or deactivation), and MCI-converter subjects demonstrating loss of deactivation (or positive activation). These levels of deactivation sit on a continuum with AD subjects and controls at the extremes.

The mechanism underlying our findings and those of other groups, implicating the PMC region in memory impairment, is not completely understood. This region is one of the first to demonstrate early hypometabolic changes in young asymptomatic subjects in their twenties and thirties who are positive for the apolipoprotein E4 allele, and are therefore at increased risk for developing AD [18]. The PMC region is also part of a cortical network revealing negative activations on PET and fMRI during a wide variety of tasks, and has been implicated in default-mode processing, supporting higher activity during passive vs. active tasks [33], [34]. EEG studies have demonstrated a similar network of regions with common spontaneous power fluctuations [33], [35], and functional connectivity fMRI studies have shown high temporal correlations in BOLD signal across these regions, implying a functional connection [36]. While fMRI, EEG, and PET data suggest medial parietal/posterior cingulate cortex involvement in early AD, it is not known if this is a primary event or a secondary consequence of MTL pathology, such as neurofibrillary tangle deposition, which occurs early on in the disease [37]. Indeed, in vivo human studies using the amyloid-binding radiotracer, C11 Pittsburgh Compound-B, have demonstrated a correlation in the posterior cortical regions between amyloid deposition, atrophy and hypometabolism, suggesting a network which is directly disrupted in early-stage AD [38]. On the other hand, MTL regions, in particular the entorhinal and perirhinal cortex, are densely interconnected to the posterior cingulate cortex, and their disruption leads to medial parietal/posterior cingulate functional metabolic changes in both animals and humans [39], [40], [41], [42]. Thus, it is also logical to hypothesize that AD-related loss of deactivation in PMC regions may potentially result from structural or functional disconnection, or both, between the PMC and MTL. This notion is strongly supported by studies with fMRI, DTI and EEG showing that a functional and structural disconnection between specific memory regions, including the PMC and medial temporal lobe, may occur in early AD [43], [44], [45], [46], [47].

MCI subjects are a heterogenous group, with some subjects undergoing progressive cognitive decline, and others remaining stable over time. The estimated annual conversion rate to AD in subjects with MCI is 10–15%. Few studies have looked at the prognostic significance of fMRI in patients with MCI. One study, by Dickerson et al., followed 32 MCI subjects over a 2.5 year clinical follow-up and found differences in extent of activation, at a given statistical threshold, in the right parahippocampal gyrus, in subjects who subsequently underwent cognitive decline, compared to those who remained stable [48]. The authors speculate that higher levels of activation in the medial temporal lobe are a compensatory adaptation to less efficient neuronal activity, due to underlying medial temporal pathology. The decliner group had worse cognitive scores compared to the stable group at baseline, and therefore might be demonstrating the effects of inefficient medial temporal lobe function. Subsequent findings of the same group demonstrate increased levels of hippocampal activation in MCI subjects compared to normal aging and AD [13]. The authors speculated that there is a phase of increased medial temporal lobe activation early in the course of prodromal AD followed by a subsequent decrease as the disease progresses. If this is the case, then single measures of medial temporal lobe activation alone would be problematic as a marker of disease activity, because there would be a period of pseudonormalization of activation that would be difficult to distinguish from normal activation.

Our study differs from this one in several respects. First, we looked at the magnitude of the fMRI response, as opposed to the extent of activation. The latter measurement is dependent upon an arbitrarily chosen statistical threshold, as well as on the length of the fMRI experiment and underlying noise in the signal. These results may therefore be difficult to reproduce in a different scanner, or using a different statistical threshold. The authors also examined differences in activation magnitude between decliners and stable MCI subjects, yet no differences were found using this index of activation. Second, in the study by Dickerson et al., MCI subjects had subjective, rather than objective memory impairment defined by cognitive testing, as opposed to our study in which objective memory impairment was an entry criterion. Thus, there is a significant difference in the samples between the two studies. Third, “Decline” was defined as an increase in CDR-SOB score by at least one point, rather than conversion to AD, as was the case in our study. We believe the latter is a more significant clinical endpoint.

There are a number of potential methodological biases that could have affected our results. For example, it has been reported in other studies that in medial parietal and frontal regions there is an early increase in activation, followed by a rapid decrease, in controls and MCI, but not AD subjects [19], [20]. Though we did not specifically study the temporal profile of activity in the PMC, we did incorporate a temporal derivative of the hemodynamic response function into our model as a covariate, and therefore believe that our measures of activation magnitude are not biased by differences in temporal shifts of the hemodynamic response function. While age may also have an effect on the shape of the hemodynamic response function [49], our MCI groups were not significantly different in age; nevertheless, to address this concern, we included age as a covariate in our ANCOVA model, and found no main effect. Lastly, variable performance on cognitive tasks has been shown to impact measured fMRI activation levels [50]. Unfortunately performance on relevant cognitive tasks tends to be inseparable from disease status and it is impossible to completely disentangle the two. To address this consideration, we ran analyses to control for differences in behavioral performance during the fMRI task across groups. Results of both ANCOVA and logistic regression analyses still demonstrated statistically significant effects of group membership, independent of behavioral performance. Again, ANOVA post hoc testing revealed a significant difference between MCI-Converter and MCI-Nonconverter groups, suggesting the prognostic value of fMRI activation, independent of task performance.

In conclusion, we have demonstrated the prognostic significance of deactivation in the PMC region, as measured by fMRI, in predicting future cognitive decline in patients with amnestic MCI. As new therapeutics for prevention and treatment of AD emerge in the coming years, fMRI may serve as an important early diagnostic and prognostic tool, along with clinical, laboratory and structural imaging measures, to identify at-risk candidates who may benefit from early intervention.
